# Applying Lipidomics to Non-Alcoholic Fatty Liver Disease: A Clinical Perspective

**DOI:** 10.3390/nu15081992

**Published:** 2023-04-20

**Authors:** Jian Huang, Giordano Sigon, Benjamin H. Mullish, Dan Wang, Rohini Sharma, Pinelopi Manousou, Roberta Forlano

**Affiliations:** 1 Liver Unit, Division of Digestive Diseases, Department of Metabolism, Digestion and Reproduction, Faculty of Medicine, Imperial College London, London W21NY, UK; 2 Department of Surgery & Cancer, Imperial College London, Hammersmith Hospital, London W21NY, UK

**Keywords:** lipidomics, NAFLD, NASH, fibrosis

## Abstract

The prevalence of Non-alcoholic fatty liver disease (NAFLD) and associated complications, such as hepatocellular carcinoma (HCC), is growing worldwide, due to the epidemics of metabolic risk factors, such as obesity and type II diabetes. Among other factors, an aberrant lipid metabolism represents a crucial step in the pathogenesis of NAFLD and the development of HCC in this population. In this review, we summarize the evidence supporting the application of translational lipidomics in NAFLD patients and NAFLD associated HCC in clinical practice.

## 1. Introduction and Aims

**Figure 1:**
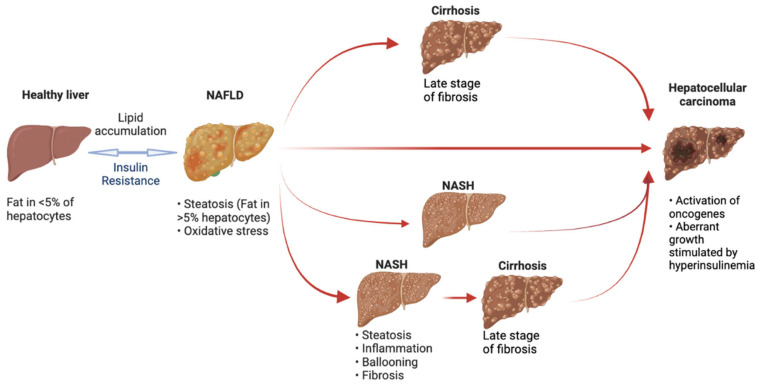
NAFLD development and progression. NAFLD develops from a healthy liver when lipid accumulates in the context of insulin resistance. NASH represents the most aggressive component of the spectrum and is associated with hepatocellular injury and hepatic inflammation. In NAFLD patients, fibrosis may develop in a variable degree up to cirrhosis, while HCC can develop in both cirrhotic and non-cirrhotic patients. (HCC: hepatocellular carcinoma; NAFLD: non-alcoholic fatty liver disease; NASH: non-alcoholic Steatohepatitis).

Hepatocellular carcinoma (HCC) is one of the most common cancers and the third leading cause of cancer-related mortality in the world [1,2]. Chronic viral hepatitis B (HBV) and C (HCV), alcohol consumption, and features of metabolic syndrome (i.e., type-2 diabetes mellitus, obesity) are well known risk factors for HCC [3]. Non-alcoholic fatty liver disease (NAFLD) represents a leading cause for liver transplantation and chronic liver disease worldwide, with an overall prevalence of 30% [4]. NAFLD is characterized by more than 5% fat accumulation [5], which results from the impaired lipid metabolism and excessive accumulation of free fatty acids (FFAs) in the hepatic tissue [6]. Fat accumulation and lipotoxicity represent a significant factor that promotes NAFLD progression to fibrosis and up to cirrhosis and HCC (Figure 1) [7]. According to recent data, the estimated incidence rate of NAFLD-associated HCC has been increasing over the past 20 years [8]. In the United States only, the incidence of HCC caused by NAFLD increased by 9% per year between 2004 and 2009 [9]. More recently, it has been estimated that the incidence rate of HCC attributed to NAFLD among all the causes of HCC has increased from 9% in 2015 to 34% in 2020 [10,11].

The alteration of lipid metabolism represents a crucial step in the development and progression of NAFLD, and it has been recognized as one of the hallmarks of oncogenesis in this population [12]. It is also known that the uncontrolled lipid metabolism provides energy for the rapid HCC proliferation and progression to metastatic disease [12,13]. Among other techniques, advancements in mass spectrometry techniques have introduced lipidomics to translational medicine and research in recent years [14,15]. Lipidomics refers to the discipline studying the lipid profile in cells, biologic fluids, and tissue [16]. Among other metabolites, lipids are not only the most abundant in body circulation, but they also exert several biological functions, such as storing energy, signaling, and acting as structural components of cell membranes [15]. In recent years, the importance of the imbalance of lipid metabolism in diseases has drawn much more attention, especially with regards to NAFLD progression and development of HCC. In this review, we discuss potential applications of lipidomics in clinical practice.

### Introduction to Hepatic Lipids

Hepatic lipids cluster in different categories, each one with a specific distribution, expression, and function, i.e., proliferation, survival, apoptosis, and drug resistance of cells [17,18,19]. The main lipid categories are: fatty acyls, glycerolipids, glycerophospholipids, sphingolipids, saccharolipids and polyketides, sterol lipids, and prenol lipids [20,21]. Overall, lipids typically have three components: one glycerol backbone, two fatty acid tails, and one phosphate group [22]. According to the Lipid Maps, the position of the double bond within the molecule determines both the structure and the function of the lipids [23,24]. Interestingly, variations in the structure of the lipids translate into different biological functions [20]. For example, the difference of one double bond between docosahexaenoic acid (22:6n3, DHA) and docosapentaenoic acid (22:5n6, DPA) results in a different function of these two lipids [24]. Docosahexaenoic acid (22:6n3, DHA) is an activator for the peroxisome proliferator-activated receptor-α (PPARα) in the liver, while the function of docosapentaenoic acid (22:5n6, DPA) in the liver is unclear [25]. In addition to the number of double bonds, the previous study on lipidomics also reveals that the position of the double bond in the lipid can also determine the level of the lipid in cancerous tissue vs. adjacent tissue [17]. Phosphocholines, phosphatidylethanolamines, and phosphatidylinositols with 2, 4, 6 double-bond show a downregulation in HCC tissues, whereas phosphocholines, phosphatidylethanolamines, and phosphatidylinositols with 0, 1, 3 double-bond show an upregulation in adjacent tissues [17].

Among others, transcriptional factors may also influence the expression and abundance of hepatic lipids. Specifically, several transcriptional factors have been identified as modulators of adipogenesis as well as of de novo fatty acid synthesis and lipogenesis. Some transcriptional factors are able to regulate the expression of intrahepatic lipids, including sterol regulatory-element binding proteins (SREBPs) and carnitine palmitoyltransferases (CPTs) [26,27,28]. The sub-species sterol regulatory-element binding proteins 1 (SREBP1) promotes acetyl-CoA carboxylase (ACC) and fatty acid synthase (FASN), which activate the de novo lipogenesis [29]. Moreover, SREBP-1 shifts glutamine and glucose metabolism towards the fatty acids synthesis, a mechanism that supplies sufficient energy and nutrition for the rapid proliferation of cells [29,30]. Another up-stream regulator, sterol regulatory-element binding proteins 2 (SREBP2), is able to stimulate cholesterol production and facilitate lipid accumulation in normal hepatocytes [31,32,33].

Carnitine palmitoyltransferases 1 (CPT1) also represents another important modulator of the hepatic lipid metabolism. CPT1, which belongs to the carnitine shuttle system, is a key rate-limiting enzyme of fatty acid oxidation in lipid metabolism, whose dysregulation can affect energy homeostasis [28]. CPT1 transfers long-chain CoA fatty acids into the mitochondria for β-oxidation, as the inner membrane of the mitochondria is impermeable to fatty acids [28]. The CPT1 enzymes can be divided into three isoforms: CPT1a, CPT1b, and CPT1c [34,35]. CPT1a is mainly liver and pancreas-specific [36]. Moreover, CPT1a can be up-regulated by a high-fat diet to promote beta oxidation [37,38], likely via the lipid-activated peroxisome proliferator-activated receptor-α (PPARα) [39].

To conclude, there is evidence suggesting that the composition and regulation of hepatic lipids may impact their biological functions in the energy homeostasis of the liver.

## 2. Lipidomics in NAFLD

### 2.1. Lipid Metabolism and Lipotoxicity in the Pathogenesis of NAFLD

The accumulation of hepatic triglycerides represents the crucial step for the development of the disease. Overall, a reduced fatty acid β-oxidation and very-low-density lipoprotein (VLDL) export are associated with the massive accumulation of fatty acids and triglycerides in the liver [40]. Subsequently, the mismatch between β-oxidation and the oxidative phosphorylation leads to oxidative stress, which, in turn, contributes to lipotoxicity, cellular damage, and fibrosis progression [41,42]. Moreover, the resulting production of reactive oxygen species (ROS) induces mitochondrial dysfunction, which, in turn, exacerbates ROS production and, ultimately, lipotoxicity [43,44]. An aberrant mitochondrial lipid metabolism contributes to the dysfunction of the electron transport chain (ETC), and it also induces the expression of Sirtuin (SIRT) 3 and the damage of mitochondrial DNA [43].

Lipotoxicity represents a crucial step in the pathogenesis of NAFLD and the progression to NASH, as it may lead to the accumulation of toxic lipids in the hepatocyte. It is also the hallmark of the diagnosis of NASH [45]. Lipotoxicity translates into organellar dysfunction, abnormal activation of signaling intracellular signaling pathways, chronic inflammation, and, ultimately, apoptosis [46,47]. The underlying mechanism involves several cellular components, such as endoplasmic reticulum (ER) stress, lysosomal permeabilization, and mitochondrial dysfunction. Specifically, histology from patients with NASH showed defective electron transport chain (ETC) function together with specific morphological alterations, such as enlarged mitochondria, rounded cristae, and alterations of the mitochondrial DNA [48]. An incomplete β-oxidation of fatty acids, such as palmitic acid, has been shown to impair mitochondrial function, as it may disrupt the ETC directly via the activation of phosphatases [45,49]. Such changes may lead to the accumulation of ROS and other toxic metabolites, such as superoxide, palmitic acid, and ceramides [50,51,52]. An increased amount of superoxide may, in turn, generate further oxidative damage and sustain both lipotoxicity and cellular membrane damage [45]. In addition to mitochondrial dysfunction, lipotoxicity may cause ER stress. For instance, a previous lipidomic study carried out on liver tissue reported that the high level of diglycerides, ceramides, phospholipids, and saturated fatty acid can directly induce the ER stress by the activation of the Unfolded protein Response (UPR) and the expression of pro-apoptotic molecules, such as B-cell lymphoma 2 (BLC2) [50,52]. Furthermore, apoptosis may be induced directly by saturated free fatty acids via both intrinsic and extrinsic pathways. The ER and the oxidative stress caused by accumulated FFAs stimulate the activation of C/EBP Homologous Protein (CHOP) and the cJUN NH2-terminal kinase (JNK) pathway. The activation of CHOP and JNK is then followed by the upregulation of more pro-apoptotic factors and the release of cytochrome C and caspase 9 [45]. Lipotoxicity has also been associated with greater intra-hepatic inflammation. For instance, toxic lipid metabolites, such as palmitate, can induce the production of pro-inflammatory factors by activated macrophages via TNF-related apoptotic factors [53]. Furthermore, it has been demonstrated that hepatocytes, under the stimulation of saturated fatty acid, may release pro-inflammatory cytokines (i.e., CXC-chemokine ligand 10 (CXCL10)), which further sustain inflammatory response and cellular damage. Finally, in patients with NASH, lipotoxicity has been associated with impaired autophagy in the form of defective phagosome formation and lysosomal acidification [54]. Specifically, a mixture of palmitic and oleic acid has been shown to inhibit autophagic flux and reduce lipophagy, contributing to the vicious circle of lipotoxicity-induced damage [55].

After being exposed to toxic lipids, injured hepatocytes release a large group of extracellular vesicles, such as exosomes, microparticles, and apoptotic bodies. These byproducts may not only perpetuate inflammation but may also elicit fibrosis by activating non-parenchymal cells [56]. Moreover, apoptotic bodies will be included by stellate cells and then active them into HSC activation, with the production of α–smooth muscle actin and collagen [57]. Some recent evidence also suggests that toxic fatty acids may be able to stimulate Kupffer cells and HSCs directly. For instance, palmitic acids induce toll-like receptor (TLR) 2 and TLR4 in macrophages and activate a pro-inflammatory response in KCs [58]. Palmitate can also elicit actin production from activated HSCs [58].

In terms of specific lipid species, phosphocholine is one of the main components of cell membranes and of lipid droplets and plays a crucial role in maintaining physiological cellular activities. Imbalances in the phosphocholine expression may result into hepatocyte dysfunction and have been associated with NAFLD development and progression [6,43,44,59]. Interestingly, there has been evidence suggesting that even changes in the structure of lipids may translate into different biological effects. For instance, an odd-chain phosphatidylcholine was reported to have a negative correlation with the progression of NAFLD [60]. In addition to the structure of lipids, the level of diversity lipids shows a close correlation with the progression from the normal liver to NAFLD. Specifically, a lower level of ceramides, a lipid species that modulates cell proliferation, and a lower level of polyunsaturated triglycerides were both previously associated with an impaired metabolism of the hepatocytes [17,61,62]. Conversely, supplementation with ceramides and polyunsaturated triglycerides was shown to have a hepato-protective effect via promoting the apoptosis of aberrant hepatocytes [63]. There has also been recent evidence suggesting that the regulation of the expression of lipids may influence the development and progression of NAFLD. For instance, PPARα knock-out mice, when starved, rapidly develop fatty liver disease, as the inhibition of CPT1a accumulates fatty acids in hepatocytes [64,65]. Moreover, SREBPs have been identified as possible oncogenes in the pathogenesis of hepatocarcinoma [26,66].

Finally, bile acids (BAs) have also been involved in the pathogenesis and progression of NAFLD. BAs are synthesized from cholesterol in hepatic tissue; thus BAs are characterized by amphipathic molecules. This unique character leads BAs to solubilize the lipid bilayer [67], which can result in the disruption of cellular structure. Therefore, the high level of intracellular BAs can increase the high risk of apoptosis and promote the infiltration of inflammatory factors [68]. Furthermore, BAs can directly interact with the gut microbiota in the intestinal compartment. Growing evidence suggests that BAs have a significant influence on the progression of NAFLD and NASH via affecting the gut microbiota to regulate the hepatic lipids [68,69]. However, the precise mechanism of the apoptosis signaling pathway induced by the BAs’ metabolism in cellular activities is unclear.

To conclude, there is evidence suggesting that both composition and regulation of hepatic lipids may impact the development and progression of NAFLD.

### 2.2. Translational Lipidomics for Diagnosing NAFLD

Overall, hundreds of lipids species in serum and hepatic tissue, triglycerides, diglycerides, sphingolipids and cholesteryl esters have shown a significant difference of species in patients with NAFLD compared to healthy controls [60,70,71,72,73,74,75,76]. The main results from lipidomic studies published in the field have been summarized in Table 1 and Table 2. A previous study using liquid chromatography mass spectrometry (LC-MS) suggested that serum levels of phosphatidylethanolamines (PE), phosphocholine (PC), and sphingomyelin (SM) were able to distinguish the patients with NAFLD from healthy controls [71]. Peng et al. identified that saturated triglycerides were increased whereas polyunsaturated triglycerides were reduced in NAFLD compared to healthy controls [60]. Consistent with Peng’s results, Gorden et al. also found that up to 15 triglycerides and 7 cholesteryl esters were up-regulated in the hepatic tissue of NAFLD patients (Table 2) [70]. Across different studies, nine lipids were consistently increased in patients with NAFLD: phosphocholine (PI)(40:5), triglyceride (TG) (52:4), diacylglycerol (DG) (34:2), and diacylglycerol(DG)(36:2) (Table 1) [60,70,71,72,75]. Along with quantitative changes of circulatory lipids, there seems to be a difference in the distribution of lipids in the liver of NAFLD patients, too. Three-dimensional studies have shown that low-density lipoprotein and very low-density lipoproteins are more abundant in the steatotic regions, whereas phosphatidylinositol and arachidonic acid prevail in the fat-sparing areas of the same livers [77].

From a clinical perspective, the lipid profile appears to be different in patients with NAFLD depending on the presence of different risk factors and genetic predisposition. Of note, in a study using LC-MS, diacylglycerol, triglyceride, and sphingomyelin were found to be significantly increased in the sera of obese patients with NAFLD compared to lean NAFLD, suggesting a direct influence of visceral adiposity [78]. Moreover, in a study using direct flow injection electrospray ionization tandem mass spectrometry (ESI–MS/MS), saturated ceramide-enriched liver lipidome was observed in those with NASH in the context of metabolic syndrome and insulin resistance, but not in those with “genetic-driven”, PNPLA3-associated NASH, i.e., those carrying I148M variant of the gene [79]. Furthermore, another recent study demonstrated that carriers of the HSD17B13 variant have increased phospholipids in their liver but have minimal fibrosis [80]. Interestingly, in this group, the presence of phospholipids was independent of hepatic insulin sensitivity. Ethnicity may also influence the lipid profile in these patients, as Hispanics were found to have higher FFA and lysophospholipids than Caucasians, indicating ethnic-related lipidomic signatures [81].

Overall, these results suggest that a distinct lipid profile reflects different combinations of metabolic risk factors and the clinical phenotype of the patient, suggesting the role of lipidomics in precision medicine.

### 2.3. Translational Lipidomics for Staging NAFLD

Despite being the gold standard for the diagnosis and staging of NAFLD, liver biopsies carry several risks, such as cost, bleeding risk, and pain for the patient [71,82]. For this reason, the research on biomarkers for NAFLD has been flourishing over the past few years. Interestingly, it has been demonstrated that alterations in the liver tissue lipidome reflect the lipid profile measurement in the plasma, opening the field for the use of a lipid profile as a biomarker for the histological features of NASH [83]. A combination of circulating lipids may improve the diagnosis and risk-stratification in patients with NAFLD and may identify those with NASH accurately [71,72]. In a group of NAFLD patients, a score combining serum lipids, assessed by nanoparticle-tracking techniques, and genetic variants was able to predict fat fraction, as measured by MRI-PDFF [84]. Moreover, in a cohort of patients with biopsy-proven NASH, phosphatidylcholine levels, assessed by LC-MS, were strongly associated with severity of ballooning [85]. In a similar study, phosphocholine (14:0/18:2) and phosphatidic acid (18:2/24:4) were positively correlated with NAS score, whereas phosphocholine (18:0/0:0) was correlated positively with the fibrosis stage [86]. Lipidomics may also be a useful tool to predict disease progression. Using the same lipidomic technique, our group has previously demonstrated that a score combining metabolic profile and lipoproteins was able to identify fast fibrosis progressors and performed better than noninvasive markers [87].

From a clinician’s perspective, cardiovascular events are the main cause of morbidity and mortality in patients with NAFLD [88]. Nevertheless, identifying those at higher risk for cardiovascular events in this population remains a challenge [89]. Interestingly, ectopic fat deposits, such as myocardial and epicardial fat, show a specific lipid composition, which is different from the hepatic one [90]. Moreover, a higher abundance of diacylglycerol and ceramide (Cer) in the ectopic fat deposits, measured by LC-MS, seems to be associated with an overall stronger lipotoxicity effect [91]. Overall, these results suggest that lipidomics may be employed to identify those at risk of MACE in this population.

Finally, lipidomic approaches may also provide more insight into metabolic changes with different treatments in patients with NAFLD. A 6-month treatment with polyunsaturated fatty acids was able to change the lipid profile of patients with NASH, resulting in an underlying lower lipogenesis, endoplasmic reticulum stress, and mitochondrial dysfunction [92]. Similarly, in patients who underwent weight loss, there was a significant decrease in circulating lysophospholipids [93]. More studies are required to evaluate how the changes in lipidomic profile may be translated into clinical events.

## 3. Lipidomics in NAFLD-Related HCC

### 3.1. Lipid Alterations in the Pathogenesis of NAFLD-Associated HCC

Lipids play a crucial role in cancer cellular activities as they are a significant part of cell membranes, signaling molecules, and pools of metabolic energy [94]. As such, the lipidomics have been regarded as a potential direction for elucidating pathogenesis and for discovering new biomarkers in patients with HCC. Specifically, the reprogramming of the lipid metabolism comes as an essential requirement in the rapidly proliferating cells, through the Warburg effect and the activation of the de novo fatty acid synthesis [95,96]. Briefly, the Warburg effect is a well-known reprogramming of the lipid metabolism in order to supply energy for the cancer cells in the form of increased glycolysis and oxidative phosphorylation [97]. Under physiological conditions, glycolysis supplies energy for nucleotide and amino acid synthesis by producing Nicotinamide adenine dinucleotide phosphate (NADPH). Under the Warburg effect, an enhanced glycolysis produces higher concentration of NADPH, which is used for the synthesis of both nucleotides and lipids for the cellular membrane [98]. Therefore, the high glycolytic rate fulfils the energy demand for the production of nucleotides and amino acids for the highly-proliferating HCC cells [98,99]. In addition to the Warburg effect, de novo fatty acid synthesis is also an important marker of lipid reprogramming in the development of HCC. Compared to normal hepatocytes, the HCC cells favor de novo fatty acid synthesis over the exogenous fatty acid sources [100].

A recent study demonstrated that ceramides were down-regulated, whereas cholesteryl ester were upregulated in the hepatic tissue of patients with NAFLD-related HCC compared to those with NAFLD without HCC [62]. Interestingly, Oskouian et al. have shown that ceramide is able to induce apoptosis in cancer cells via the suppression of their cellular metabolism [19]. Specifically, the signaling pathway of apoptosis can be inhibited by consuming ceramide or complex glycosphingolipids (GSL) [101].

### 3.2. Translational Lipidomics in the Management of Patients with NAFLD-Associated HCC

NAFLD is a growing cause of HCC worldwide, with almost one third of the diagnosis of HCC made in the absence of cirrhosis [102]. With many cases of HCC being diagnosed at a more advanced stage, there is an unmet need for biomarkers for an earlier diagnosis in this population.

Comparing the lipidome from patients with NAFLD-related HCC versus NAFLD and healthy controls may provide useful insight into improving HCC prediction in these patients. Lipidomic profile carried out by Lu et al. in 257 patients (including 113 NAFLD patients and 144 NAFLD-HCC patients) was able to distinguish NAFLD from NAFLD-HCC patients [103]. A subsequent lipidomic study using LC-MS, conducted by Lewinska et al., described a distinct lipidomic profile in patients with NAFLD-HCC compared to those with alcohol and viral associated HCC [104].

Recent mass spectrometry studies have also suggested that with the HCC advancing, there is a progressive increase in triglyceride concentration and a decrease in the polyunsaturated triglyceride concentration [17]. Interestingly, the progressive depletion in polyunsaturated fatty acids has been explained by an upregulation of fatty acid transporters in NAFLD-HCC tumors [104]. Moreover, two very long chain fatty acids, lignoceric and nervonic acid, were not detected in patients with HCC compared to those with NAFLD [105]. Of note, lignoceric and nervonic acids are strictly associated with liver homeostasis and may have a role as mediators in limiting the intrahepatic inflammation in NAFLD [105]. Interestingly, a combination of biochemical features and serum lipids was able to predict the presence of HCC with 97% accuracy [104]. In addition, Lin et al. also showed that a decrease in palmitic acyl-based glycerophospholipids was associated with metastatic HCC [106]. However, more studies are required to explore the use of applied lipidomics as predictors of metastatic disease. Finally, regarding the down-regulation of ceramide with the progression of NAFLD to HCC [107], a vinca alkaloid drug, Vinblastine, has been reported to increase the level of ceramide in hepatic tissue and to inhibit progression to HCC [108]. Therefore, focusing on ceramide or ceramide-related genes might be the next target for the therapy of NAFLD-HCC. Despite these encouraging observations, future work will need to focus on the role of lipidomics as predictors of responses to treatment in patients with HCC.

## 4. Conclusions

Increasing evidence supports the application of lipidomic techniques to precision medicine when assessing patients with NAFLD and NAFLD-associated HCC. Nevertheless, more studies are required to evaluate the applicability of these techniques to clinical practice.

## Figures and Tables

**Table 1:** Results from published studies on circulatory lipids in patients with NAFLD.

References	Plasma		Serum	
	Increased	Reduced	Increased	Reduced
Peng et al., (2018) and Gorden et al., (2015) [60,70]	PI(40:5) [60,70]			
Peng et al., (2018) and Perakakis et al., (2019) [60,71]		PC(40:8) [60,71] LPE(16:0) [60,71]		
Gorden et al., (2015) and Perakakis et al., (2019) [70,71]	TG(52:4) [70,71] DG(34:2) [70,71] DG(36:2) [70,71] DG(36:3) [70,71] PC(36:4) [70] PI(36:1) [70]	PC(36:4) [71] PI(36:1) [71]		
Gorden et al., (2015) and Mayo et al., (2018) [70,72]	TG(50:2) [70] TG(52:1) [70] TG(54:5) [70]		TG(50:2) [72] TG(52:1) [72] TG(54:5) [72]	
Gorden et al., (2015) and Loomba et al., (2015) [70,75]	20-COOH AA [75] 5-HETE [75] 15-HETE [75] 11,12 diHETrE [70,75] 14,15 diHETrE [70,75] 19,20 DiHDPA [75]	20-COOH AA [70] 5-HETE [70] 15-HETE [70] 12,13 diHOME [70,75] 9,20DiHDPA [70]		

PC: phosphocholine; PI: phosphatidylinositol; LPE: lysophosphatidylethanolamine; TG: triglyceride; DG: diacylglycerol; 20-COOH AA: 20-carboxy arachidonic acid; 5-HETE: 5-hydroxyeicosatetraenoic acid; diHETrE: dihydroxyeicosatrienoic; DiHDPA: dihydroxydocosapentaenoic.

**Table 2:** Results from published studies on intrahepatic lipids in patients with NAFLD.

References	Tissue	
	Increased	Reduced
Peng et al., (2018) and Gorden et al., (2015) [60,70]	TG(48:1) [60,70] TG(48:2) [60,70] TG(48:3) [60,70] TG(49:1) [60,70] TG(50:1) [60,70] TG(50:4) [60,70] TG(50:3) [60,70] TG(51:2) [60,70] TG(48:0) [60,70] TG(50:0) [60,70] TG(50:2) [60,70] TG(51:1) [60,70] TG(52:1) [60,70] TG(52:2) [60,70] TG(52:4) [60,70] CE(16:0) [60,70] CE(16:1) [60] CE(18:1) [60,70] CE(18:2) [60,70] CE(18:3) [60,70] CE(20:3) [60,70] CE(20:4) [60,70] CE(22:5) [60,70] CE(22:6) [60] LPC(18:0) [60]	CE(16:1) [70] CE(22:6) [70] LPC(18:0) [70]
Peng et al., (2018) and Gorden et al., (2011) [60,73]	DG(30:0) [60,73] DG(32:1) [60,73] DG(32:2) [60,73] DG(38:5) [60,73] PE(38:4) [60]	PE(38:4) [73]
Peng et al., (2018) and Chiappini et al., (2017) [60,76]	Cer(42:1) [60]	
Gorden et al., (2011) and Gorden et al., (2015) [70,73]	DG(34:3) [73]	DG(34:3) [70]
	DG(36:0) [73]	DG(36:0) [70]
	DG(36:5) [73]	DG(36:5) [70]
	DG(38:0) [73]	DG(38:0) [70]
Gorden et al., (2015) and Chiappini et al., (2017) [70,76]		PC(38:4) [70,76]
Gorden et al., (2011) and Chiappini et al., (2017) [73,76]	PC(36:1) [73]	PC(34:1) [73,76]
	PI(38:5) [73]	PC(36:1) [76]
	PS(36:1) [73,76]	PI(38:5) [76]
Peng et al., (2018) and Gorden et al., (2015) and Gorden et al., (2011) [60,70,73]	DG(32:0) [60,70,73]	
	DG(34:0) [60,70,73]	
	DG(34:1) [60,70,73]	
	DG(34:2) [60,70,73]	
	DG(38:6) [60,70,73]	
	DG(36:1) [60,70,73]	
	DG(36:2) [60,70,73]	
	DG(36:3) [60,70,73]	
	DG(36:4) [60,70,73]	
Gorden et al., (2011) and Gorden et al., (2015) and Chiappini et al., (2017) [70,73,76]	PS(34:1) [70,73,76]	PC(36:4) [70,73,76]

PC: phosphocholine; PI: phosphatidylinositol; PS: phosphatidylserine; PE: phosphatidylethanolamine; Cer: ceramide; TG: triglyceride; DG: diacylglycerol; CE: cholesteryl ester; LPC: lysophosphatidylcholine.

## Data Availability

Data is contained within the article.
